# Edible Mushrooms as a Potential Component of Dietary Interventions for Major Depressive Disorder

**DOI:** 10.3390/foods11101489

**Published:** 2022-05-20

**Authors:** Agata Fijałkowska, Karol Jędrejko, Katarzyna Sułkowska-Ziaja, Marek Ziaja, Katarzyna Kała, Bożena Muszyńska

**Affiliations:** 1 Department of Pharmaceutical Botany, Faculty of Pharmacy, Jagiellonian University Medical College, Medyczna 9, 30-688 Kraków, Poland; karol.jedrejko@gmail.com (K.J.); katarzyna.sulkowska-ziaja@uj.edu.pl (K.S.-Z.); kat3kala@gmail.com (K.K.); muchon@poczta.fm (B.M.); 2 Department of Histology, Faculty of Medicine, Jagiellonian University Medical College, Kopernika 7, 31-034 Kraków, Poland; marek.ziaja@uj.edu.pl

**Keywords:** *Cordyceps*, major depressive disorder, diet, *Hericium erinaceus*, Reishi, serotonin

## Abstract

Dietary interventions for people suffering from major depressive disorder (MDD) are an ongoing field of research. In this article, we present a comprehensive background for understanding the possibility of using edible medicinal mushrooms as an adjunctive treatment for MDD. We start with a brief history of MDD, its diagnosis, epidemiology and treatment, and the effects of diet on depression symptoms, followed by a review of neurobiological, behavioral, and clinical studies of medicinal mushrooms. We specifically highlight the results of preclinical and clinical studies on dietary supplementation with three selected mushroom species: Lion’s mane (*Hericium erinaceus*), Caterpillar mushroom (*Cordyceps militaris*), and Lingzhi/Reishi (*Ganoderma lucidum*). Preliminary small-sample clinical studies suggest that Lion’s mane can influence well-being of humans. In the case of Reishi, the results of clinical studies are equivocal, while in the case of Caterpillar Mushroom, such studies are underway. Edible mushrooms contain 5-hydroxy-L-tryptophan (5-HTP), which is a direct precursor of serotonin—a neurotransmitter targeted in pharmacotherapy of MDD. Therefore, in light of the well-recognized role of stress as a pathogenic factor of MDD, we also describe the neurobiological mechanisms of the interaction between stress and serotonergic neurotransmission; and summarize the current state of knowledge on dietary supplementation with 5-HTP in MDD.

## 1. Introduction

### 1.1. Definition

For at least 2500 years, depression has been recognized as a maladaptive, prolonged reaction to adverse circumstances that have detrimental psychosocial implications [1]. This approach—initiated by Hippocrates—was dimensional, since melancholy was viewed as an exaggeration of naturally occurring sadness. Therefore, for evaluating the severity of the condition, a physician had to contextualize the symptoms within personal history and take into account the seriousness of potential causes [1]. In the early 20th century, depression was classified into two categories: melancholic and neurotic depression [1]. The former was believed to be caused by unknown brain damage and considered a more serious condition, while the latter was thought to have a psychosocial origin and not require hospitalization; this assessment method is often referred to as “etiological”. The first editions of the Diagnostic and Statistical Manual (DSM) of the American Psychiatric Association published in the 1950s and 1960s described melancholic depression as a type of psychosis and neurotic depression as a defense mechanism against anxiety. Additionally, the existence of different forms of neurotic depression was well established in the clinic, but until the late 1970s, the exact number and characteristics remained a widely debated topic among scientists [1].

Although the above debate was far from conclusion, and necessary research was still lacking, the third edition of the DSM published in 1980 described a single category of major depressive disorder (MDD), which was defined solely based on symptoms [2]. In the fifth and most recent edition of the DSM, MDD is still defined as a unitary entity characterized by decontextualized symptoms, which include either: (1) depressed mood or (2) loss of interest or pleasure (anhedonia); and at least five out of nine other symptoms (e.g., fatigue, insomnia, suicidal thoughts, diminished concentration, and psychomotor delay) [3]. To qualify as a major depressive episode, the symptoms have to persist for a minimum of 2 weeks and cause significant distress and disturbances in initiating and performing daily activities [3]. Individuals with recurrent episodes are diagnosed with MDD.

### 1.2. Epidemiology

The World Health Organization estimated that, in 2015, 322 million people were living with MDD around the world, which constituted 4.4% of the global population [4]. The disease mainly affects women, with 5.1% of females worldwide suffering from it, compared to 3.6% of the male population [4]. Moreover, a higher incidence of MDD in females is observed across studied age groups and regions. Among psychiatric disorders, MDD is the leading cause of suicide, accounting for approximately 33% of 800,000 suicidal deaths each year [5]. Depression is also the leading cause of nonfatal health loss as measured by Years Lived with Disability (YLD). It has been estimated that MDD accounted for 7.5% of global YLD in 2015, with low- and middle-income countries bearing >80% of the burden of this disease [4].

Compared to other affective disorders, depression has a low heritability rate of ~37% [6]; for instance, the rate of heritability of bipolar disorder is estimated to be between 60% and 85% [7]. Furthermore, genome-wide association studies [8] and transcriptome-wide association studies [6] have, respectively, identified 44 and 94 genes associated with an increased risk of MDD, a majority of which are not specific to this condition, but rather predispose to global vulnerability [7]. One of the most important environmental factors that interact with MDD vulnerability throughout the lifespan is stress [9]. However, modulation of stress hormones is not necessary for achieving desired clinical outcomes in MDD patients [10]. This points to the complexity of the relation between the etiology and treatment of MDD, which—due to both the chronic nature of this disease and the proven impact of early life adversity on the disease risk—are separated by plastic changes in the brain [11,12].

### 1.3. Treatment

A recent study investigating the effectiveness of over 140 available pharmacological and nonpharmacological MDD treatments identified two evidence-based treatment modalities, namely the use of second-generation antidepressants (ADs) and cognitive behavioral therapy (CBT) [13]. Although not conclusive, the report may provide clinicians and patients with valuable guidance, as there are at least 87 known psychological and 56 interventions derived from alternative medicine, aimed at treating MDD [13]. Second-generation ADs are a group of nearly 15 drugs introduced in the 1980s and 1990s, and most of them are either selective serotonin reuptake inhibitors (SSRIs) or serotonin and noradrenaline reuptake inhibitors (SNRIs). CBT—a combination of behavioral and cognitive therapies—emerged around the same time as SSRIs and SNRIs. In principle, this approach to psychotherapy focuses on modifying cognitive distortions and behavioral patterns, in order to regulate emotions and develop strategies that can help the patient to cope with potential triggers. The efficacy of second-generation ADs and CBT is comparable, and the treatment effects are small (for example, Hedge’s g for ADs is −0.35 [13]). Therefore, it is not uncommon to combine these two approaches, especially due to the fact that SSRI-only treatment leads to full remission in about one-third of MDD patients [14]. Evidence suggests that a combination of ADs with CBT is more effective than ADs alone, resulting in moderate-to-medium effect sizes (Hedge’s g for ADs and CBT is 0.49) [15]. However, this effect may be restricted to adults, since SSRI-only treatment is as effective as SSRI–CBT combination in the case of children and adolescents [16]. It also seems that a significant proportion of the one-third of MDD patients who are treatment-resistant to the abovementioned therapies [14] will benefit from esketamine administered alone [17,18] or as an adjunct to other treatments [19].

## 2. Monoaminergic Neuromodulation, HPA, Inflammation and MDD

MDD is characterized by a multitude of symptoms accompanied by structural changes in both cortical (orbitofrontal, cingulate, insular, and temporal cortex) [11] and subcortical (hippocampus, amygdala) [12] areas that regulate the interaction between affective and cognitive processing. The orbitofrontal cortex, cingulate cortex, hippocampus, and amygdala are considered parts of the limbic system, which plays a major role in regulating motivation and emotions. Through their connections with the hypothalamus, another limbic structure, these regions also control the endocrine and autonomic nervous system, while functioning under the neuromodulatory influence of the ascending monoaminergic systems from the brainstem. The main neurotransmitters of monoaminergic systems are serotonin, dopamine, and norepinephrine, which regulate several brain functions such as mood, attention, reward processing, sleep, appetite, and cognitive abilities [20]. The role of monoamines in MDD is thought to be related to the action of ADs, which inhibit their reuptake from the synaptic cleft and/or increase their accumulation.

The role of serotonin in MDD has been most widely studied. It has been shown that experimental reduction in the amount of tryptophan, a precursor of serotonin, leads to a recurrence of acute symptoms of MDD in patients who were cured to remission [21]. In addition, a decrease in the number of serotonin receptors was observed in various brain structures in patients [22]. However, the mechanism leading to a decrease in the amount of serotonin in patients with MDD remains unknown, because studies of its metabolites in the blood or urine are equivocal.

The course of MDD may also involve changes in the noradrenergic system, such as reduced metabolism of noradrenaline, a decrease in the density of its receptors, and increased activity of tyrosine hydroxylase decomposing it at locus coeruleus—the main source of noradrenaline in the brainstem [23]. Clinical data also indicate the efficacy of noradrenaline reuptake inhibitors in the treatment of MDD [24].

Increasingly, similar to bipolar disorder, evidence highlights the role of dopamine in MDD [25]. MDD patients have been found to have reduced dopamine transmission as well as decreased concentration and uptake of dopamine transporter [26]. In addition, more than half of people with Parkinson’s disease, who are characterized by degenerated dopamine projections to the striatum, show symptoms of MDD before the onset of symptoms related to the musculoskeletal system [27].

The hypothalamus regulates the endocrine and autonomous system through the hypothalamic–pituitary–adrenal (HPA) axis. This neuroendocrine system is responsible for the stress response, which is one of the factors contributing to MDD [9]. The HPA axis acts as a negative feedback loop, where: stressors elicit the production of corticotropin-releasing hormone (CRH) from the hypothalamus; CRH causes secretion of adrenocorticotropic hormone (ACTH) from the pituitary; ACTH stimulates the secretion of cortisol from the adrenal cortex; and cortisol reversibly inhibits the secretion of ACTH. In some MDD patients, cortisol was found to be significantly increased, and this level decreases to normal as the disease subsides [10]. The administration of synthetic cortisol causes inhibition of cortisol secretion in healthy people. However, in a significant number of MDD patients, this negative feedback is disrupted, and cortisol remains at a consistently high level [10], suggesting impaired HPA regulation, resulting in abnormal and prolonged stress response. Symptoms similar to those of MDD were also observed in animals that were experimentally administered CRH to the nervous system. These animals exhibited autonomic system responses in the form of increased heart rate, increased blood pressure, or reduced digestion, accompanied by a change in behaviors such as sleep and wakefulness, rhythm disorders, decreased sex drive, or increased anxiety [28]. In MDD patients, increased content of CRH in the cerebrospinal fluid and reduced mRNA expression of the CRH receptor in the frontal cortex have been observed [29,30].

The activity of HPA can be disturbed by an inflammatory response of the immune system. Pro-inflammatory cytokines such as IL-1, IL-6, and TNF-α activate the HPA and cause the so-called “sickness behavior” syndrome, which is characterized by similar symptoms as MDD, such as fatigue, psychomotor slowdown, anhedonia, or cognitive impairment [31]. In animals, blockade of pro-inflammatory processes leads to effects similar to those observed with the administration of ADs [32]. In addition, pro-inflammatory cytokines influence the metabolism of monoamine neurotransmitters, mainly serotonin, through indoleamine 2,3-dioxygenase, which is responsible for the breakdown of tryptophan and the formation of neurotoxic kynurenine [33].

## 3. Diet and MDD

Numerous studies have investigated the possible relationship between dietary patterns and MDD [34,35]. Although the first meta-analyses of observational studies on this subject suggested an inverse relation between a healthy diet and depression symptoms [36,37], the results were inconclusive as most of the analyzed studies were cross-sectional. Since data obtained in a single time-point cannot allow for determination of whether dietary patterns are a risk factor, concomitant, or an effect of MDD, Molendijk et al. (2018) reviewed only longitudinal observational studies [38]. The authors mainly found an association of high-quality or low pro-inflammatory diet with lower levels of depression symptoms, but not with clinical diagnosis of MDD [38]. Moreover, the lack of association between low-quality diet and incidence of MDD suggested that consumption of low-quality foods is concurrent to MDD, and not a risk factor of this condition [38]. These results were replicated in a recent meta-analysis, which differentiated cross-sectional and longitudinal studies, as well as dietary measures [34]. Overall, a traditional Mediterranean diet, as well as a lower Dietary Inflammatory Index, was associated with a lower risk of incident depression (i.e., a major depressive episode with no prior MDD history) in longitudinal studies [34]. Since the associations of dietary patterns with depression symptoms have been shown to be explained by confounding factors, such as socioeconomic status and physical activity [39], it is important to note that studies controlling these factors suggest diet as being independently associated with the risk of incident depression (see Table 1 in [34] for detailed list of studies and controlled factors). An outstanding question is whether these associations hold true in low- and middle-income countries, as these were underrepresented in the meta-analysis [34].

Due to its low risk and potential beneficial effect in reducing the severity of MDD symptoms, a high-quality diet may seem a valuable preventive strategy. Indeed, in the PREDIMED trial, a subsample of 620 patients with type 2 diabetes who were at a high risk of cardiovascular disease had a 40% lower risk of incident depression after at least 3 years of following the Mediterranean diet supplemented with nuts [40]. On the contrary, in the more recent randomized clinical trial (MooDFOOD) conducted among overweight adults, no effect of diet on the risk of a depressive episode was observed after 21 behavioral therapy sessions aimed at the improvement of dietary patterns (or multinutrient supplementation) over the course of 1 year [41]. Thus, it is still unclear which groups of patients and/or healthy individuals could benefit from preventive dietary interventions. It is therefore intriguing that data from 15 studies in patients suffering from comorbid, subclinical depression, or depressive symptoms secondary to other disease revealed that dietary treatment had slightly (Hedge’s g = 0.162) positive effects on depression symptoms [42]. Of note, these effects were mainly observed among female participants [42].

A recent review of preclinical animal studies identified at least nine possible biological factors contributing to the effects of diet on the symptoms of MDD. These factors include inflammation, oxidative stress, gut microbiota, HPA axis, adult neurogenesis and brain-derived neurotropic factor (BDNF), tryptophan-kynurenine metabolism, mitochondrial dysfunction, epigenetics, and obesity [43]. Other notable factors identified based on clinical observations as possibly mediating the abovementioned effects are chronic diseases comorbid with MDD, such as metabolic syndrome, type 2 diabetes, or cardiovascular disease [43]. Although there are no experimental studies in humans in this regard, it has been shown that no differences in the adaptation of brain metabolism to metabolic stress (fasting) were observed between healthy controls and MDD patients [44] and that women with MDD history displayed higher post meal blood pressure than those without MDD history [45]. Some studies have examined supplementation with isolated nutraceuticals as an adjunct to pharmacotherapy. A meta-analysis of these studies showed that S-adenosylmethionine (SAMe), methylfolate, omega-3, and vitamin D produced positive results [46].

## 4. 5-Hydroxy-l-tryptophan and MDD

l-tryptophan is an exogenous amino acid that acts as a precursor for the synthesis of serotonin in a metabolic pathway involving two enzymes: tryptophan hydroxylase (TPH) and aromatic amino acid decarboxylase (DDC). 5-Hydroxy-l-tryptophan (5-HTP), a product of TPH and an immediate precursor of serotonin, is broken down by DDC. Contrary to its precursors, serotonin does not cross the blood–brain barrier, and therefore its total pool in the brain is determined by the amount of substrates and the activity of TPH, which is the rate-limiting step for serotonin synthesis. The main source of serotonin in the brain is neurons of nine raphe nuclei located in the medial brainstem [47]. The axons of these neurons project to the majority of the brain, with rostral raphe nuclei mainly sending their serotonergic projections to the forebrain and caudal group to the lower brainstem and spinal cord [47].

Experimental studies in rats have clearly shown that different forms of stressors (e.g., tail shock, forced swimming, loud sound stress) activate TPH specifically in one of the nuclei of the rostral group—the median raphe nucleus [48]. Serotonergic projections of this nucleus directly target basal ganglia and hippocampi, and indirectly target septum and cingulate cortex [47], which are structures involved in the processing of and response to affective stimuli. Following stress, the activity of TPH is upregulated, refilling the intracellular stores of serotonin, which are depleted due to the increased firing of serotonergic neurons, mediated by glucocorticoids [48]. Moreover, daily changes in the expression of TPH2 (one of the two TPH-encoding genes expressed predominantly in the brain) are also dependent on daily rhythms of glucocorticoids, which are regulated by the suprachiasmatic nucleus [48]—the main pacemaker of the brain located in the hypothalamus. Although initial reports indicated that chronic stress does not influence TPH2 expression in the rodent brain [48], a recent study suggested that this is dependent on sex in the case of humans. Specifically, a higher transcription of TPH2 in the dorsolateral prefrontal cortex was observed in female, but not in male MDD patients, when compared to healthy controls of the same sex [49]. This may indicate that in women with MDD the neuronal response to stress is abnormal, which would lead to a higher demand on TPH2 activity due to stress-induced depletion of serotonin stores. Interestingly, a previous meta-analysis of studies addressing the deleterious effects of tryptophan depletion on verbal episodic memory, which is a function contingent on dorsolateral prefrontal cortex activity [50], showed that women are generally more prone to acute lowering of tryptophan levels [51].

Recent meta-analyses of studies comparing plasma tryptophan concentrations between MDD patients and healthy controls yielded ambiguous results. A study of 24 published datasets combined with authors’ own data showed a significantly higher decrease in tryptophan levels in unmedicated MDD patients, and a weak correlation between the severity of MDD symptoms and tryptophan concentrations [52]. On the other hand, a more recent meta-analysis with more stringent inclusion criteria, which included nine studies, did not find any significant effect of MDD on peripheral tryptophan levels [33]. Similarly, the results of meta-analyses of studies examining the therapeutic potential of tryptophan [46] or 5-HTP [53] in MDD are inconclusive, with 5-HTP found to be a more promising treatment option for the disease.

The above discrepancies in results might be related to the substantial heterogeneity of such studies, most of which lack placebo-controlled designs—an issue that has been raised for the last 20 years by authors analyzing the subject [53,54]. In the case of tryptophan, another confounding factor might be its second—and primary—kynurenine metabolic pathway, which has been proposed to play a role in inflammation-induced MDD [55]. Specifically, two metabolites resulting from kynurenine transformation in microglia have been shown to either exhibit neurotoxicity (3-hydroxykynurenine) or activate *N*-methyl-d-aspartate receptors (quinolinic acid) [33], the antagonists of which (e.g., esketamine) are effective against treatment-resistant MDD [56]. It still remains unclear whether tryptophan metabolism shifts away from serotonin to kynurenine in MDD, and the available results are conflicting [57,58]. Perhaps, the different subtypes of MDD (melancholic, anxious, energy-related) are associated with different profiles of tryptophan metabolites [59].

As a direct precursor of serotonin, 5-HTP is devoid of or has a low potential to cause undesirable effects in humans. There are no reports indicating serotonin syndrome, and only moderate gastrointestinal symptoms have been observed at a wide dose range [60]. Moreover, pilot experiments examining the effect of administration of 5-HTP at doses of 200–300 mg/day as an adjunct to pharmacological treatment with different classes of ADs have shown promising results in treatment-resistant MDD patients [60]. Such effects are explained by a synergistic effect of 5-HTP and serotonin transporter (SERT) inhibitors on extracellular serotonin levels measured directly in preclinical studies [61] or indirectly (via effects on cortisol levels) in humans, where the addition of 5-HTP can cause a 4-fold increase in the physiological effects of an SSRI [62].

To allow SERT inhibitors to achieve their therapeutic effects in MDD patients, it is necessary to not only elevate the extracellular levels of serotonin but also sustain the increase over the course of the day; it has been shown that tryptophan depletion causes a relapse in ~50% of remitted MDD patients within hours—an effect that may be exacerbated in females and chronically ill patients treated with SSRIs [21]. For this reason, most of the ADs have a half-life of >20 h, and a single missed dose of an SSRI may lead to a discontinuation syndrome [60]. Due to its half-life of 2 h, the dosing regimen of 5-HTP in humans can be challenging, and it is estimated that even three doses a day would cause 5-fold fluctuations in daily levels of 5-HTP (compared to 0.3-fold fluctuations of SSRIs) [60]. Therefore, for adjunctive 5-HTP therapy to work, either a slow-release formulation or dietary sources of the compound are needed.

Several edible mushroom species have so far been identified, with relatively high content of 5-HTP (Table 1). Of note, the highest content was found in all species of *Pleurotus*, which is popular in vegetarian cuisine. Moreover, high yields of mushroom mycelia can be easily achieved in standardized in vitro conditions by using bioreactors, and this mode of production can possibly boost levels of 5-HTP compared to fruiting bodies (Table 1).

**Table 1:** Bioactive indole compounds found in selected 15 genera of edible mushrooms. NA—not available.

Mushroom	Form	Bioactive Indole Derivatives Compound Concentration [mg/100 g]					References
		Serotonin	l-Tryptophane	5-HTP	Tryptamine	Melatonin	
*Agaricus bisporus* (White bottom mushroom)	Fruiting bodies	5.21	0.39	<0.001	0.06	0.11	[63]
*Armillaria mellea* (Honey mushroom)	Fruiting bodies	2.21	4.47	<0.001	2.74	<0.001	[64]
*Boletus badius* (Bay bolete)	Fruiting bodies	0.52	0.68	<0.001	0.47	<0.001	[64]
*Boletus edulis* (King bolete)	Fruiting bodies	10.14	0.39	0.18	1.17	0.68	[64]
*Cantharellus cibarius* (Chanterelle)	Fruiting bodies	29.61	0.01	0.02	0.01	0.14	[63]
*Ganoderma applanatum* (Bracket fungus)	Mycelium	NA	1.76	<0.001	1.12	0.02	[65]
*Ganoderma lucidum* (Reishi)	Mycelium	10.58	NA	NA	NA	0.98	[65]
*Hericium erinaceus* (Lion’s mane)	Mycelium	NA	NA	152.72	11.88	1.04	[66]
	Fruiting bodies	NA	NA	92.19	1.19	<0.001	[66]
*Lactarius deliciosus* (Saffron milk cap)	Fruiting bodies	18.42	<0.001	0.25	<0.001	1.29	[63]
*Laetiporus sulphureus* (Chicken of the wood)	Mycelium	NA	14.08	1.5	1.16	<0.001	[65]
*Leccinum rufum* (Birch bolete)	Fruiting bodies	31.71	<0.001	0.02	1.05	0.08	[63]
*Leccinum scabrum* (Rough-stemmed bolet)	Fruiting bodies	13.99	9.56	<0.001	<0.001	<0.001	[67]
*Lentinula edodes* (Shitake)	Fruiting bodies	1.03	0.58	24.83	0.04	0.13	[67]
*Macrolepiota procera* (Parasol mushroom)	Fruiting bodies	<0.001	3.47	22.94	0.92	0.07	[67]
*Pleurotus citrinopileatus* (Golden oyster mushroom)	Mycelium	<0.001	7.82	368.67	3.71	<0.001	[68]
	Fruiting bodies	<0.001	13.84	128.89	1.29	<0.001	[68]
*Pleurotus djamor* (Pink oyster mushroom)	Mycelium	<0.001	24.34	703.56	<0.001	<0.001	[68]
	Fruiting bodies	7.68	24.84	193.95	3.54	<0.001	[68]
*Pleurotus eryngii* (King trumpet mushroom)	Mycelium	8.54	7.60	221.51	2.67	0.08	[68]
	Fruiting bodies	13.18	35.28	149.73	17.84	0.13	[68]
*Pleurotus florida* (Pearl oyster mushroom)	Mycelium	<0.001	<0.001	215.53	<0.001	0.09	[68]
	Fruiting bodies	3.31	10.84	95.21	1.52	<0.001	[68]
*Pleurotus ostreatus* (Oyster mushroom)	Mycelium	<0.001	1.89	120.11	1.03	4.45	[68]
	Fruiting bodies	6.52 NA	<0.001 5.79	2.08 67.45	0.91 1.04	<0.001 0.33	[68]
*Pleurotus pulmonarius* (Indian oyster)	Mycelium	<0.001	17.29	553.87	<0.001	<0.001	[68]
	Fruiting bodies	<0.001	11.85	117.02	<0.001	<0.001	[68]
*Suillus bovinus* (Jersey cow mushroom)	Fruiting bodies	<0.001	25.90	15.83	3.15	<0.001	[67]
*Suillus luteus* (Slippery Jack)	Fruiting bodies	34.11	2.61	1.63	<0.001	0.71	[69]
*Trametes versicolor* (Turkey tail)	Mycelium	NA	3.91	0.9	1.69	0.01	[65]
*Tricholoma equestre* (Man on horseback)	Mycelium	0.59	1.03	0.34	0.59	0.32	[70]
	Fruiting bodies	0.18	2.85	0.58	2.01	<0.001	[70]

## 5. Medicinal Mushroom Species and MDD

### 5.1. Hericium erinaceus

**Figure 1:**
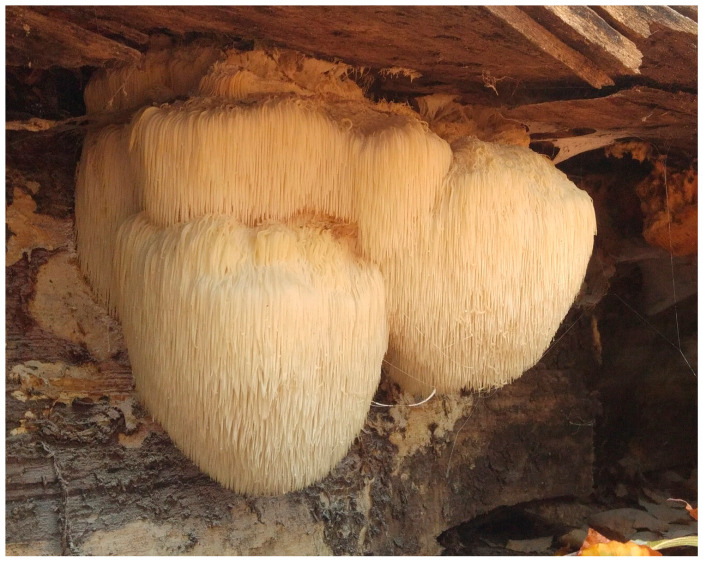
*Hericium erinaceus* in its natural habitat (photo by Paweł Stasiowski).

*Hericium erinaceus* (common names: lion’s mane and yamabushitake, Figure 1), an edible and medicinal mushroom species, is widespread across East Asia, endangered with extinction in Europe, and grown commercially in USA and Asia. Its medicinal properties have been known for several ages, as evidenced by its use in traditional Asian folk medicine [71]. The bioactive compounds identified in *H. erinaceus* include indole, fenol, steroid, and lactone compounds, terpenoids (erinacines, hericerins, and hericenones, Table 2), alkaloids (hericirine), polysaccharides, and glycoproteins [71]. Both in vitro and in vivo studies have shown that these compounds act as antioxidants and immunomodulants, and may also display antitumor, antidiabetic, and neuroprotective effects [71]. Recent studies have proven further biological effects of *H. erinaceus*, which include defense against mitochondrial dysfunction [72], as well as against high levels of stress hormones [73]; potential positive impact on gut microbiota in rodents [74], and humans [75]; and elevation of connectivity in the developing rodent brain [76]. Thus, this species seems to modulate six out of nine mechanisms that could mediate the impact of diet on MDD [43] (Table 3).

To date, three preclinical [77,78,79] and three clinical [80,81,82] studies have investigated the potential influence of *H. erinaceus* on affective functions. In a 2010 study on a small sample of 12 women undergoing menopause, Nagano et al. observed that 4-week supplementation with 2 g/day of powdered fruiting bodies of *H. erinaceus* caused a significant reduction in self-reported depression symptoms and indefinite complaints to levels that were—however—indistinguishable from the placebo group (*n* = 14), in which the reduction was also present, but statistically nonsignificant [81]. This effect could not be driven by erinacines (for chemical structure, see Table 2), the only compounds proven to date with the ability to cross the blood–brain barrier [83,84], as they are found only in the mycelium and not in the fruiting bodies of *H. erinaceus* [85]. Interestingly, Okamura et al. (2015) observed higher norepinephrine turnover (measured by free 3-Methoxy-4-hydroxyphenylglycol, i.e., MHPG levels in saliva), without any effect on self-reported somatic, anxiety, or depression symptoms in eight women (no placebo group was included in the study) after 4 weeks of supplementation with 585 mg/day of hericenone [82]. Finally, in a subgroup of 15 obese individuals with high self-reported depression and anxiety symptoms, Vigna et al. (2019) found that administration of 1.5 g/day of *H. erinaceus* (1.2 g mycelium, 0.3 fruiting body) for 8 weeks caused a reduction in both anxiety and depression symptoms [80].

A case study showed that an 86-year-old male MDD patient treated with fruiting bodies for 6 months was cured to remission [86]. These results constitute a promising body of preliminary evidence for the potential use of *H. erinaceus* as a dietary supplement in patients with affective disorders. However, large-scale clinical trials are needed. Currently, a low-sample (n = 40 participants per arm) phase II trial is being carried out to determine the efficacy of 6-month supplementation with *H. erinaceus* as an adjunct to pharmacotherapy with SSRIs or SNRIs (ClinicalTrials.gov identifier: NCT04179006). The symptoms will be measured eight times with the 17-item Hamilton Rating Scale for Depression. However, due to the poor retest reliability of some items of the scale [87] and difficult interpretation [88], the 6-item version of the questionnaire would be more preferable [89]. Nevertheless, the results of this trial—and similar others—are valuable.

Recently, preclinical research on the potential neurobiological effects of *H. erinaceus* in the context of MDD was extensively reviewed [85,90]. The behavioral studies mainly used the Tail Suspension Test (TST) or Forced Swimming Test (FST) to measure the stress-coping strategy of rodents [91]. In general, ADs change the behavior of animals from immobility (passive stress-coping) to swimming and/or climbing (active stress-coping), while factors that may be pathogenic in MDD (e.g., chronic stress, inflammation) have an opposite effect. Yao et al. (2015) reported that, in mice, even single p.o. administration of the same formulation as used by Okamura et al. (2015) in women, but at a ~12-fold lower dose than the daily dose used in the human study, caused reversal of passive stress-coping induced by a proinflammatory agent (lipopolysaccharide, LPS) [77]. Of note, in the same experimental set-up (i.e., mice challenged with LPS and tested in TST and FST), the authors observed the effects of ADs administered in at least ~10 higher doses than the daily human dose [92], which is common in pharmacological studies on mice. Both Ryu et al. (2017) and Chiu et al. (2018) observed similar behavioral results as Yao et al. (2015) with chronic (4 weeks) supplementation with ethanolic extract of *H. erinaceus* at doses ranging from 60 mg/kg/day in healthy mice [80] and from 200 to 400 mg/kg/day in animals subjected to restrain stress for 14 days [79]. For reference, these doses were 2–10 times higher than those used in aforementioned human studies investigating the effects of *H. erinaceus* on depression symptoms [81,82].

In vivo studies in mice also confirmed two of the six mechanisms that could be associated with the effects of *H. erinaceus* on depression symptoms, which include anti-inflammatory effect in the periphery [78] as well as in the hippocampus [79], and neurogenic and prosurvival effects on hippocampal neurons [79,80]. In addition, mushroom supplementation (400 mg/kg/day) was found to restore monoamine (serotonin, dopamine, noradrenaline) neurotransmission in the hippocampus of stressed animals [79], which suggests that *H. erinaceus* may be a valuable adjunct to pharmacotherapy with SSRIs and SNRIs.

**Table 3:** Summary of mechanisms implicated in the impact of diet on depression symptoms with confirmed effects in preclinical rodent studies.

***Herricium erinaceus***			
Biological effect	Neuroprotective effect	Effects in preclinical in vivo studies	Reported effects in clinical studies
Anti-inflammatory	Erinacine-A promotes neuronal survival in mouse hippocampus via BDNF and NFκB increase in response to LPS [78].	Reduction in passive stress-coping induced by LPS [78]. Decrease in plasma proinflammatory cytokines: TNFα [77,78], and Il-6 [78]. Increase of plasma anti-inflammatory cytokine Il-10 [77].	Reduction in self-reported depression [80,81] and anxiety symptoms [80]. Higher noradrenaline turnover [82]. Modulation of gut microbiota [75].
Antioxidative	Neuroprotective against DEHP [72] and high corticosterone levels [73] via antioxidative and antiapoptotic activity in vitro.	*no data*	
Gut microbiota	*no data*	Polysaccharides regulate inflammation in the gut via microbiota [74].	
HPA axis	Neuroprotective against high corticosterone levels in vitro [73].	Reversal of passive stress-coping induced by repeated restraint stress in mice [78]. Prevents a decrease in noradrenaline, serotonin and dopamine in hippocampi of stressed mice [78].	
Mitochondria protection	Neuroprotective against DEHP-induced mitochondrial dysfunction in vitro [72].	*no data*	
Neurogenesis and BDNF	Erinacine-A increases proliferation of hippocampal progenitors in the subgranular zone of the dentate gyrus [79] and increases via BDNF and NFκB signaling [78].	Reduction in passive stress-coping compared to non-supplemented mice [79].	
***Cordyceps militaris***			
Biological effect	Neuroprotective effect	Effects in preclinical in vivo studies	Reported effects in clinical studies
Anti-inflammatory	*no data*	Cordycepin normalized hippocampal IL-6 and TNFα levels increased by chronic stress in mice [93], and serum IL-1β in chronically stressed rats [94]	*no data*
Antioxidative	*no data*	Increase in brain antioxidant levels in rats [94].	
HPA axis	*no data*	Reversal of passive stress-coping and consummatory anhedonia induced by chronic unpredictable mild stress in mice [93] and rats [94,95]. Recovery of noradrenalin, dopamine, serotonin and glucocorticoid receptor levels in the hypothalamus of chronically stressed rats [95].	
Neurogenesis and BDNF	*no data*	Cordycepin slightly upregulated hippocampal BDNF levels decreased by chronic stress in mice [93].	
***Ganoderma lucidum***			
Biological effect	Neuroprotective effect	Effects in preclinical in vivo studies	Reported effects in clinical studies
Anti-inflammatory	*no data*	Polysaccharides normalized hippocampal proinflammatory (Il-6, TNFα) and anti-inflammatory (Il-10) cytokine levels increased by chronic stress in mice [96].	Improvement [97] or worsening [98] of self-reported fatigue and improvement of well-being [96,98]. Reduction [99] or no change [98] in depression and anxiety symptoms.
HPA axis	Polysaccharides are neuroprotective against high corticosterone levels in vitro [100].	Polysaccharides reverse passive stress-coping and consummatory anhedonia induced by chronic unpredictable mild stress in mice [97,100].	
Neurogenesis and BDNF	Triterpenes promote neuronal survival via NGF and BDNF signaling in vitro [101].	Polysaccharides restore hippocampal [97] and frontal cortex [100] BDNF levels decreased by chronic stress in mice.	

### 5.2. Cordyceps *spp.*

**Figure 2:**
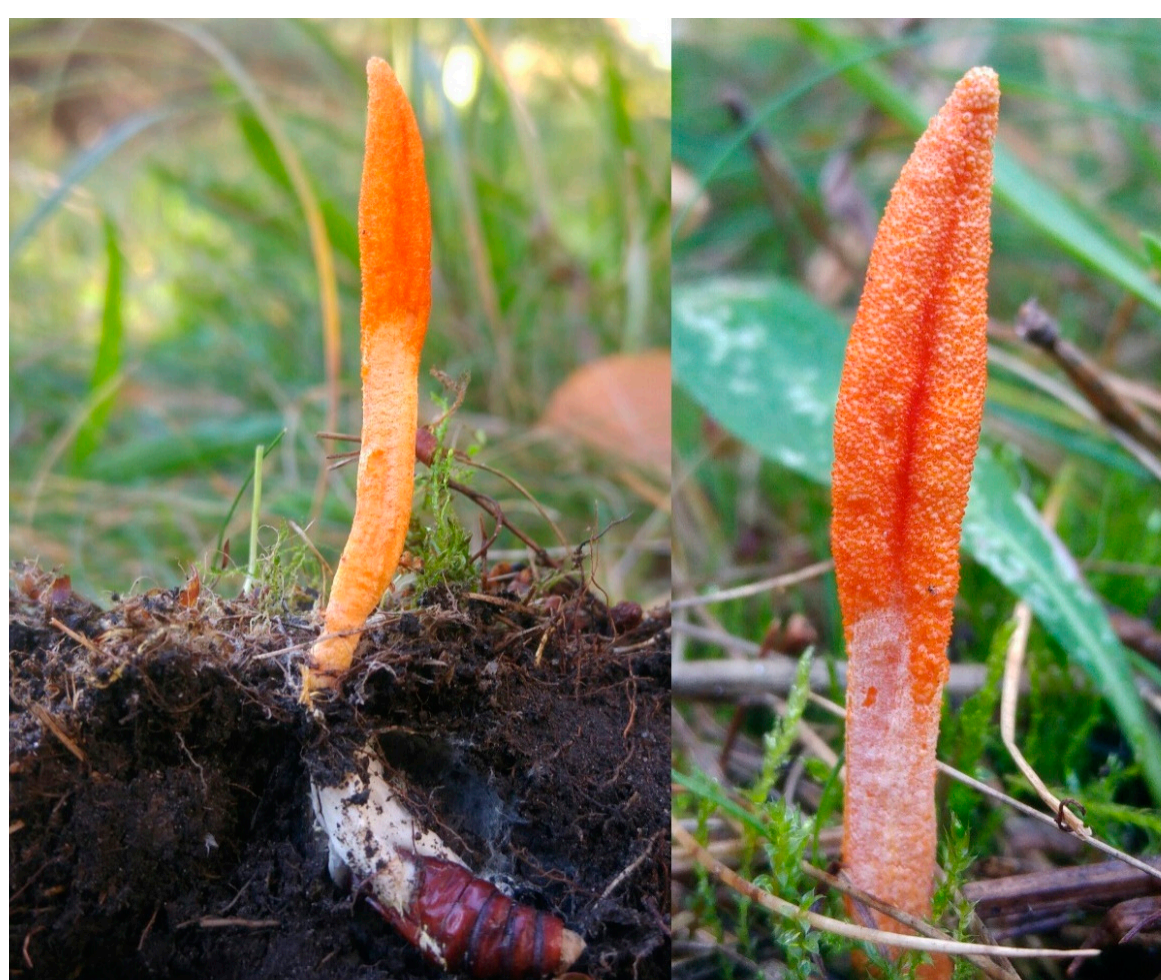
*Cordyceps militaris* in its natural habitat (photo by Paweł Stasiowski).

*Cordyceps* spp. (phylum: *Ascomycota*) have been used in traditional Chinese medicine due to its immunostimulating effect, antifatigue properties, and beneficial effects against respiratory diseases (Figure 2) [93].

The fruiting bodies and mycelium of *C. militaris* contain various bioactive compounds, including nucleosides (cordycepin; for chemical structure, see Table 2), polysaccharides, amino acids (γ-aminobutyric acid, ergothioneine), and phenolic compounds (phenolic acids and flavonoids).

Several in vivo studies on rodents have investigated the effects of *Cordyceps* on physiological, neurobiological, and behavioral changes induced by stress [93,94,95,102,103,104,105,106,107]. Koh et al. (2003) observed changes in the weight of four glands (thymus, spleen, and adrenal and thyroid glands) in rats that were supplemented with a hot water fraction of *Cordyceps sinensis* mycelia for 8 days, and reported that the supplement counteracted the effects of 2 days of stress in the tested animals [103]. Of note, the authors found that *C. sinensis* boosted swimming endurance in mice [103], which suggests that all behavioral studies that rely on the timing of a physical activity requiring exertion, such as FST and TST, should be interpreted with caution.

Nishizawa et al. (2007) compared the effects of 5-day supplementation with hot water and superficial fluid extracts of *C. sinensis* in TST, and concluded that even 10-fold higher doses of hot water extract than that used by Koh et al. (2003) did not have an influence on the mobility of mice [107]. However, the superficial fluid extract caused an increase in mobility when administered at 5 and 10 mL/kg, without any effect on spontaneous locomotor activity in the open field test [104], which suggests that the TST results should be interpreted in terms of a switch from passive to active stress-coping. Moreover, the authors explored the involvement of three monoaminergic neurotransmission systems (i.e., dopaminergic, noradrenergic, and serotonergic) in the observed effects. They found that α1-adrenergic and D2-dopaminergic receptor antagonists (prazosin and sulpiride, respectively) attenuated the effect of 10 mL/kg superficial fluid extract in the TST—a result that was not observed after the inhibition of serotonin synthesis by p-CPA [104]. The conclusion drawn based on TST that *C. sinensis* did not act via serotonergic neurotransmission was strengthened by the lack of effects of the extract on 5-HTP-induced head twitch response [104].

Since mushrooms can absorb and accumulate trace metals, Wang et al. (2011) examined the effect of 4-week administration of vanadium-enriched *C. sinensis* on stress-coping in diabetic rats [95]. In line with the findings of Nishizawa et al. (2007), a study showed that treatment with *C. sinensis* reversed the effect of streptozotocin-induced diabetes and returned passive-coping (immobility) to healthy control levels through climbing behavior [95], which is mediated by noradrenergic neurotransmission [105]. However, rats treated with *C. sinensis* also spent twice as much time as healthy controls in active stress-coping via swimming [95], which is mediated by serotonergic neurotransmission [105]. Moreover, the observed effects could not be attributed to vanadium, as the metal itself had no effect in modified FST [95]. Unfortunately, the authors did not control for the possible effects of *C. sinensis* on spontaneous locomotor activity, which—if elevated—would point to the stimulating effect of the mushroom extract, or perhaps restoration of normal metabolism in diabetic animals, rather than an influence on stress-coping. Such an interpretation would explain the contradictory results regarding the involvement of the serotonergic system in the effects of *C. sinensis*. Indeed, evidence from several rodent [106,107,108] and human [109,110] studies indicates the ability of *Cordyceps* spp. to counteract the physiological effects of exertion/effort. The only other study investigating the effect of *Cordyceps* mushrooms on streptozotocin-induced diabetes in the context of stress-coping was performed with *Ophiocordyceps formosana* [111]. The results of the study showed no effect on locomotor activity in diabetic mice, but restoration of active stress-coping, accompanied by an increase in the levels of dopamine and serotonin in the hippocampus and frontal cortex [111]. Since both *C. sinensis* and *O. formosana* have common bioactive compounds, it can be assumed that the doubling of swimming time in the modified FST observed by Wang et al. (2011) was indeed mediated by serotonergic neurotransmission.

This hypothesis is supported by research on the isolated effects of one of the bioactive compounds of *Cordyceps* spp.—cordycepin. Tianzhu et al. (2014) compared the effects of 21-day p.o. administration of 20 and 40 mg/kg cordycepin with 15 mg/kg fluoxetine in mice with chronic unpredictable mild stress [93]. Similar to fluoxetine, at both doses, cordycepin reversed the behavioral effects of stress in the sucrose preference test (measuring consummatory anhedonia), open field test (measuring anxiety-like behaviors), TST, and FST [93]. In addition, the treatment induced an anti-inflammatory response, rescued the levels of BDNF, and most importantly, upregulated 5-HT2A (serotonergic receptor) protein levels, and serotonin (and dopamine) neurotransmitters’ levels in the hippocampi of stressed mice [93]. Interestingly, similar behavioral and biochemical results for dopaminergic and serotonergic neurotransmission in the hypothalamus [95] and frontal cortex [94] were observed in rats subjected to chronic unpredictable mild stress and subsequently treated with a parasitic fungus commonly found on *C. sinensis* [95] or with the water extract of *C. militaris* [112].

Li et al. (2016) investigated the effect of cordycepin on active stress-coping, and observed that subchronic (3 days) i.p. injections of 5–12.5 mg/kg did not influence the locomotor activity of mice, while inducing active stress-coping after both acute and subchronic (5 days) treatment in TST and FST [112]. The authors also noted that acute cordycepin increased the levels of AMPA-glutamatergic receptor in the prefrontal cortex, by inducing phosphorylation at the S845 site of the GluR1 subunit of the receptor—an effect that was also observed in the hippocampus after subchronic treatment [112]. Recently, Li et al. (2021) confirmed that *C. militaris* supplementation had an impact on an intracellular signaling pathway (ROCK2/PTEN/Akt) in the rodent prefrontal cortex [113], which is known to converge on targets leading to phosphorylation at the S845 site of the GluR1 subunit [114].

In summary, preclinical in vivo studies on *Cordyceps* mushrooms and their bioactive compounds have proven that they affect at least four out of nine mechanisms (i.e., inflammation, oxidative stress, HPA function, and neurogenesis; Table 3) that could be associated with the effects of diet on depression [43]. More clinical studies are however needed to verify the antidepressive potential of *Cordyceps* mushrooms. Currently, a triple-blind randomized controlled trial is ongoing in Taiwan with an aim of evaluating the impact of *C. militaris* supplementation on self-reported mood and levels of blood cortisol in adults with lowered mood (defined by a score of 8–17 in the 21-item Hamilton Rating Scale for Depression; https://clinicaltrials.gov/ct2/show/NCT04002219 (accessed on 3 March 2022)).

### 5.3. Ganoderma lucidum

**Figure 3:**
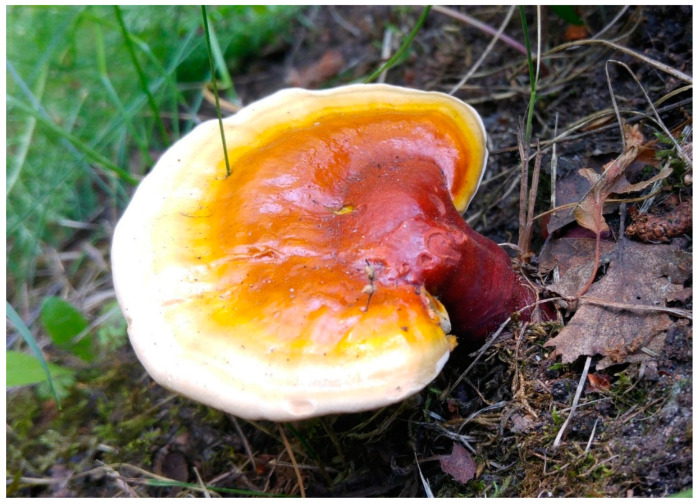
*Ganoderma lucidum* in its natural habitat (photo by Paweł Stasiowski).

The major bioactive compounds found in *Ganoderma lucidum* (common names: Reishi in Japan, Ling-Zhi in China, Figure 3) are polysaccharides and triterpenes (ganoderic acids, lucidenic acids) [115]. Chemical analyses of this mushroom species also revealed the presence of alkaloids and phenol compounds [115]. This species has been used in Asian folk medicine as an antidiabetic, antihypertensive, and antitumor agent [115]. Triterpenes, which are responsible for these effects, account for 0.244–0.444% of dried fruiting bodies and 0.555% of dried mycelium [101]. The concentration of triterpenes in the dried fruiting bodies of *G. lucidum* has been estimated at a range of 2.44–4.44 mg/g and in dried mycelium at around 5.55 mg/g, with the highest amounts of triterpenes found in the antlered form of the mushroom (“deerhorn lingzhi,” 5.87–7.03 mg/g) [101]. The concentration of polysaccharides in in vitro-produced *G. lucidum* was estimated at around 1.5 mg/g DW, and the content of polysaccharides has been shown to be successfully increased up to ~40% by genetically manipulating the metabolic pathways of the mushroom [116].

Research on the mechanisms involved in the functioning of the nervous system has shown that triterpenes isolated from *G. lucidum* can selectively inhibit acetylcholinesterase with moderate affinity to the protein [117], as well as exhibit neuroprotective effects in vitro [118]. However, to date, no in vivo studies investigating stress reactivity have been performed on triterpenes isolated from *G. lucidum*. Since both water extracts of mycelium [117] and alcohol extracts of the fruiting bodies of *G. lucidum* [97] have been shown to reduce passive stress-coping of rodents in FST, it seems worthwhile to conduct further studies on these extracts. On the other hand, studies on polysaccharides from *G. lucidum* conducted in chronically stressed mice have not only confirmed their abovementioned behavioral effects but also shown their ability to decrease serum corticosterone levels [96], modulate glutamatergic neurotransmission, BDNF levels, and immunoreactivity in the hippocampus [99], as well as modulate serotonergic and noradrenergic neurotransmission, BDNF levels, and synaptic plasticity in the prefrontal cortex [96].

Interestingly, the only clinical study performed on a modest sample size confirmed the positive effects of *G. lucidum* on the affective well-being of humans treated with a mushroom extract with 25% standardized content of polysaccharides [98]. In this randomized, double-blind, placebo-controlled study of 123 Chinese patients suffering from neurasthenia, 8-weeks dietary supplementation (5.4 g/day) caused a reduction in fatigue and an improvement in well-being in 51.6% of participants (n = 62), compared to 24.6% of patients (n = 61) in the placebo group [118]. Although similar results were observed in a pilot clinical trial investigating the effect of 4-week treatment with *G. lucidum* spore powder (3 g/day) on depression and anxiety scores of 22 breast cancer survivors [119], a longitudinal study in the same population showed no effect of *G. lucidum* on the psychological well-being of 2440 former cancer patients after 6 months and 1501 survivors after 36 months [120]. Additionally, breast cancer survivors who included *G. lucidum* fruiting bodies in their diet reported a worsening of their physical well-being and an improvement of their social well-being [120], but since this was not an experimental study, causal conclusions could not be drawn. Lastly, in a randomized, double-blind, placebo-controlled pilot study of 26 women suffering from fibromyalgia, 6-week administration of *G. lucidum* (6 g/day) was not found to be more effective than placebo (n = 24) in improving life-satisfaction scores [121].

In summary, preclinical in vivo studies carried out to date have shown that *G. lucidum*-derived polysaccharides modulate at least three out of nine mechanisms (i.e., inflammation, HPA function, and neurogenesis, Table 3) associated with the effects of diet on depression [43]. Human studies on this topic are still sparse, with available results being equivocal, perhaps due to differences in the studied material and/or patient populations. Currently, no clinical trials have been registered.

## 6. Future Directions

Apart from the abovementioned need for more reproducible data on the effects of mushroom supplementation in humans, future studies should aim at identifying and comparing the active compounds of medicinal mushrooms. The pharmacokinetics and pharmacodynamics of these substances should be described, and mechanical/biological and behavioral effects tested in animal models. This effort should pave the way for preclinical and clinical studies into the interaction of biologically active compounds derived from medicinal mushrooms with psychoactive drugs—a direction inspired by promising research on substances derived from other food groups [46].

## 7. Conclusions

Evidence supporting the efficacy of a high-quality diet in mitigating the symptoms of MDD in the general population is still lacking, but it is believed that some groups of people might benefit from selected diets, such as the Mediterranean diet [34]. Although healthy eating patterns have been shown to reduce depression symptoms secondary to other diseases [42], it is unclear whether they could be considered as a preventive strategy [41] or as an adjunct to psychotherapy and pharmacotherapy. Isolated nutraceuticals have been shown to exert potential synergistic effects [46]. Preliminary studies suggest that 5-HTP combined with ADs can be an effective option for treatment-resistant MDD patients [60].

**Figure 4:**
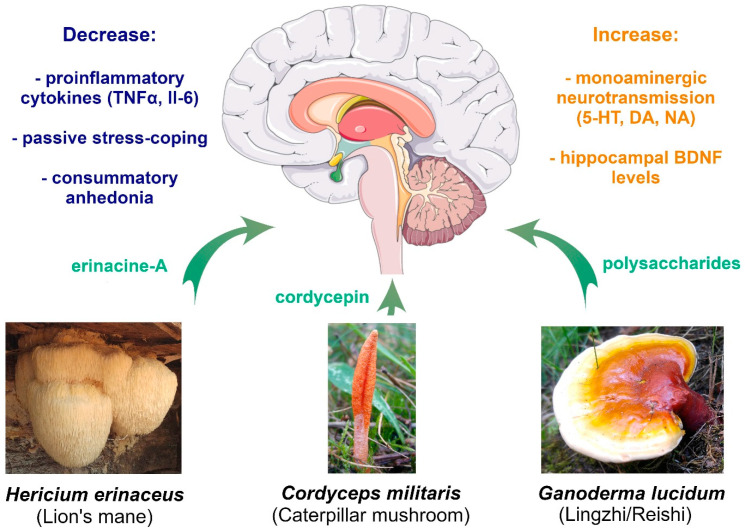
Common neurobiological mechanisms and behavioral effects observed in chronic stress rodent models of MDD after supplementation with compounds from *Hericium erinaceus*, *Cordyceps militaris* or *Ganoderma lucidum* (Figure composed using Servier Medical Art: http://smart.servier.com/(accessed on 15 May 2022)). Photos were kindly provided by Paweł Stasiowski.

Selected edible mushrooms, such as *Pleurotus* spp., are rich in 5-HTP (Table 1). Additionally, some edible medicinal mushrooms produce compounds that have been shown to affect stress-coping strategies in laboratory animals, as well as influence the neurobiological mechanisms (Figure 4), such as inflammation, HPA function, and neurogenesis, which are potentially associated with the effects of diet on MDD. These compounds include polysaccharides (e.g., those derived from *G. lucidum*), nucleosides (e.g., cordycepin), and terpenoids (e.g., erinacines) (Table 3). The efficacy of dietary supplementation with edible mushrooms in MDD patients remains ambiguous, and given the complexity of the disorder(s), small effect sizes reported in studies on the effect of diet on depression symptoms, the possibility of synergistic effects with pharmaco-/psychotherapy, significant differences in the psychometric quality of commonly used depression questionnaires, and potential ease of accessibility to edible mushrooms in the general public, experimental clinical studies should be designed with care, in order to provide reliable information.

## Figures and Tables

**Table 2:** Chemical structures of bioactive compounds found in edible mushrooms.

Name of the Compound	Chemical Structure	Occurrence in Mushrooms
L-Tryptophan	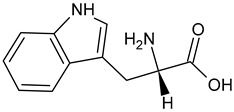	Commonly found in edible mushrooms.
5–Hydroxy-L-tryptophan	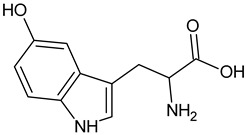	Commonly found in edible mushrooms.
Serotonin	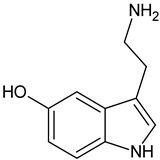	Commonly found in edible mushrooms.
Erinacine S	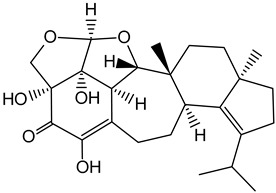	*Hericium erinaceus*
Cordycepin	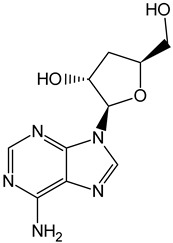	*Cordyceps militaris*
Methyl ganoderate A acetonid	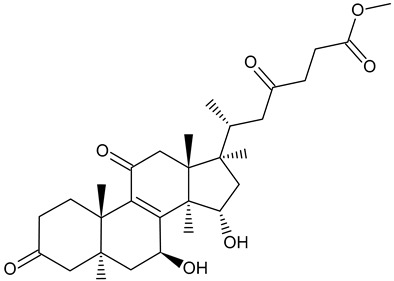	*Ganoderma lucidum*
n-Butyl ganoderate H	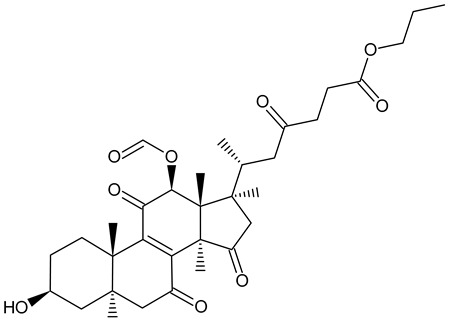	*Ganoderma lucidum*
